# Autobiographical Memory: A Scoping Meta-Review of Neuroimaging Data Enlightens the Inconsistencies Between Theory and Experimentation

**DOI:** 10.3390/brainsci15050515

**Published:** 2025-05-18

**Authors:** Edoardo Donarelli, Cristina Civilotti, Giulia Di Fini, Gabriella Gandino, Alessia Celeghin

**Affiliations:** Department of Psychology, University of Turin, Via Verdi 10, 10124 Turin, Italygiulia.difini@unito.it (G.D.F.);

**Keywords:** autobiographical memory, episodic memory, neuroimaging, scoping meta-review, semantic memory

## Abstract

**Background/Objectives:** Autobiographical memory (AM) is typically viewed in terms of comprising episodic (EAM) and semantic (SAM) components. Despite the emergence of numerous meta-analyses, the literature on these constructs remains fragmented. We aimed to summarize neural activations and to discuss the relations between constructs based on theory and experimentation, while evaluating the consistency between literature sources and discussing the critical issues and challenges of current research. **Methods:** We conducted a scoping meta-review on AM, EAM, and SAM based on meta-analytic studies in five scientific databases (PubMed, Web of Science, Scopus, PsychInfo, and PsychArticles). No temporal or language limits were applied. **Results:** We included twelve meta-analyses on AM, EAM and SAM in healthy populations. The meta-analyses of AM and EAM actually investigated the same construct, leading to misinterpretation. The two available meta-analyses on SAM used two different operationalizations of the construct. Neural data about EAM were analyzed via mean rank classification, finding the most relevant areas in the posterior cingulate cortex, hippocampus, precuneus, temporo-parietal junction, angular gyrus, and medial prefrontal cortex. SAM was linked to the posterior and anterior cingulate cortexes, middle and inferior frontal gyri, thalamus, middle and superior temporal gyri, inferior frontal and fusiform gyri, and parahippocampal cortex. **Conclusions:** Variability in reported activation patterns persists, reflecting differences in methodology and assumptions. We propose the homogenization the notations of EAM and AM based on experimental practice. In this notation, AM does not have a separate experimental task nor activation pattern and may not indicate a separate construct but an array of its components.

## 1. Introduction

### 1.1. Background and Rationale

Autobiographical memory (AM) refers to memories of ourselves, our lives and interactions with people, objects, and all aspects of the world around us [1]. These memories embrace the whole lifespan and refer to both specific events and self-related information, influencing the self in constructing who we are, who we have been, and who we expect or hope to be in the future [1,2]. On a gradient that extends from the simplest to the most complex forms of memory, autobiographical memory is certainly on the most complex side. This memory construct is typically studied using neuroimaging techniques to record changes in neural activity while participants are asked to recall a memory from their personal past, usually using a cue (e.g., [3]). These methods are often referred to as “autobiographical methods” [4] and renounce experimental control on the encoding phase (situated at any time in the participants’ past life) in order to preserve the ecological value of the material recalled by the participant. Thus, the memories studied are not about simple and inevitably impersonal items presented by the experimenter, but memories formed in response to the participants’ own non-linear and meaningful life experiences, which are more about feelings, goals, and their relations to the self [5].

This part of the complexity gradient of memory has also been studied using a different method, often referred to as “laboratory-based” [4,6]. This method involves holding experimental control over the encoding phase by giving participants information (often lists of words) that they are then asked to recall. In one commonly used procedure, i.e., the remember/know paradigm, participants are asked whether they actually “remember” the item or whether they “know” that the item was presented, without recalling the experience of mentally traveling back to the moment when the item was perceived (e.g., [7]). Responses of the first type are linked to episodic memory, which is memory of specific events, whereas responses of the second type are linked to semantic memory, which refers to more general and abstract information [8,9,10,11]. These two memory systems are conceived as distinct but largely intertwined [12].

Some researchers working primarily with laboratory methods became interested in autobiographical methods. Indeed, these methods evoke personally relevant experiences in participants [13,14], providing a more vivid sense of self in subjective time [9,10]. Furthermore, autobiographical methods allow researchers to examine memories from all stages of life, not just those acquired in the relatively short period of time between item presentation (encoding in the laboratory) and subject testing.

The idea of a distinction between more specific and more general and/or conceptual memory types was also found useful in the study of autobiographical memory, to the point that these types of memory have been considered functionally independent [15], although they are often combined in contributing to real-life memories [5]. Two resulting autobiographical memory constructs, termed episodic autobiographical memory (EAM) and semantic autobiographical memory (SAM) [15,16,17,18,19,20], have been investigated using autobiographical methods. EAM contains specific personal information related to autobiographical events, definite in time and place, whereas SAM contains semantic personal information such as general events (repeated and/or extended) and general knowledge of personal facts [15,19].

Over the past 20 years, the contemporary neuroscientific literature on AM, EAM, and SAM has evolved considerably. An ever-increasing number of studies has led to the production of a relatively large number of meta-analyses. Each of these aims to summarize evidence quantitatively by pooling the results of many single studies, trying to reduce variability in the literature towards a more definite direction [21,22,23]. While meta-analyses collect and analyze single primary studies, scoping meta-reviews collect, compare, and review high-quality summaries of the evidence such as meta-analyses [24] to continue this process of summarization and review of the literature. As research on AM and related constructs is traditionally fragmented, it is novel and valuable to systematically compare summarizations at the highest level of evidence to map the literature and enlighten areas of consistency and/or inconsistency between perspectives, experimentation, and neuroimaging data.

### 1.2. Objectives

We performed a scoping meta-review comparing neuroimaging meta-analyses on AM, EAM, and SAM in healthy individuals. Our objectives were to summarize neuroimaging data related to these constructs, evaluate agreement among sources, clarify construct relationships, and assess congruency and coherence.

A preliminary search conducted in April 2023 and updated in January 2024 confirmed no existing meta-reviews on this topic.

## 2. Materials and Methods

### 2.1. Protocol Registration and Methodology

The protocol for this scoping meta-review is registered with the Open Science Framework (https://osf.io/26hbd/ (accessed on 15 May 2025)). We followed the Joanna Briggs Institute (JBI) scoping review guidelines [25], built upon the original methodological framework proposed by Arksey and O’Malley [26]. This review report was written using the Preferred Reporting Items for Systematic Reviews and Meta-Analyses Extension for Scoping Reviews (PRISMA-ScR; [27]), see in Supplementary Materials.

In addition, the methodology integrates aspects related to the particular materials under review. In many research branches throughout the scientific literature, the increasing number of reviews and meta-analyses recently has led to the development of a methodology called scoping meta-review (e.g., [15]). This methodology applies and adapts the scoping review method [26,28] to the reviewing of different types of reviews. Different researchers include different types of reviews among narrative, scoping, and systematic reviews and/or meta-analyses [24,29,30]. We decided to focus on meta-analyses because we aimed to use the quantitative results reported to summarize the available evidence on neural activations related to our constructs of interest (AM, EAM, and SAM) (see also research questions below). Scoping reviews are used to map the literature on specific areas of research, aiming to identify and summarize key concepts, as well as potential gaps or problematic aspects [28]. The scoping meta-review format was found to be the most appropriate for this scientific investigation as it can explore, less narrow research questions while using a systematic, transparent, and reproducible a priori research protocol [24,31].

### 2.2. Ethical Approval Statement

This study is a scoping meta-review that synthesizes data from previously published meta-analyses. As no new data were collected from human participants and no direct interaction with participants occurred, ethical approval was not required. All included studies were previously published in peer-reviewed journals and are assumed to have obtained ethical approval in accordance with their respective institutional guidelines.

### 2.3. Research Questions, Eligibility Criteria, and Search Strategy

Our objectives inform the following research questions: (a) What are the neuroimaging data related to AM, EAM, and SAM in healthy individuals? (b) What is the level of agreement between the meta-analyses on neural activations and construct definitions? (c) Do the theoretical relations between constructs (EAM and SAM being the components of AM) reflect how they are studied in the literature?

The eligibility criteria reflect the research questions and inform the search strategy (and the following study selection process): (I) study design, construct studied, and neuroimaging technique(s)—only meta-analyses of AM, EAM, and/or SAM that included only studies using PET and/or fMRI; (II) population—only healthy individuals who had not received psychoactive drugs, training, psychotherapies, or other experimental manipulations of the subjects’ psychophysiological status; and (III) whole-brain focus, i.e., all papers focusing on single brain areas will be excluded.

The search strategy reflects the research questions and the eligibility criteria, and it is based on the construct(s) of interest—i.e., AM, EAM, and/or SAM; the study design, i.e., meta-analysis; and the neuroimaging method, i.e., positron emission tomography (PET) or functional magnetic resonance imaging (fMRI). To obtain documents for this scoping meta-review, we searched five scientific databases in May 2023 (PubMed; Web of Science; Scopus; PsychInfo; and PsychArticles). Individual searches are listed in Appendix A.

### 2.4. Study Selection

After elimination of duplicates, a first round of screening was conducted, evaluating titles and abstracts using the eligibility criteria described above (I, II, and III). The remaining records were screened using full texts, applying the eligibility criteria in consecutive order, i.e., applying criterion I and then evaluating the remaining records using criterion II, etc. The records that were found eligible in all rounds of screening were ultimately included. The study selection process was performed independently by two researchers (E.D. and G.D.F.) and disagreements were resolved with the help of a third researcher (A.C.).

### 2.5. Data Charting

In accordance with the JBI guidelines [25], the data extraction process was conducted with an iterative procedure, where additional relevant information to extract was identified through discussion about the data extracted up until that point. From the included studies, the following information was ultimately extracted: author(s); year; neuroimaging technique(s) used in the single studies included in the meta-analysis; memory construct(s) investigated; total number of participants of the single studies included in the meta-analysis; construct definitions used for AM, EAM, and SAM; method used to conduct the meta-analysis; neuroimaging template used; neural activity (neuroimaging data) associated with the constructs; and neuroimaging contrasts used to collect the neuroimaging data in the studies included in the meta-analysis. The data were extracted by one researcher (E.D.) and another researcher verified the correctness and comprehensiveness of the data (C.C.).

### 2.6. Data Analysis and Reporting of Results

Guided by the research questions, relevant extracted data were organized in tables used to draw a map of the meta-analytic literature. Afterwards, meta-analyses were compared based on the extracted data to discuss and summarize the neuroimaging evidence associated with each construct and the level of agreement on this evidence, also focusing on any critical issues at the methodological or theoretical level emerging from the comparison. Additionally, the analysis was also conducted to discuss how the findings inform theoretical relations between constructs and the methods used to study them in the literature.

## 3. Results

### 3.1. Study Inclusion

**Figure 1:**
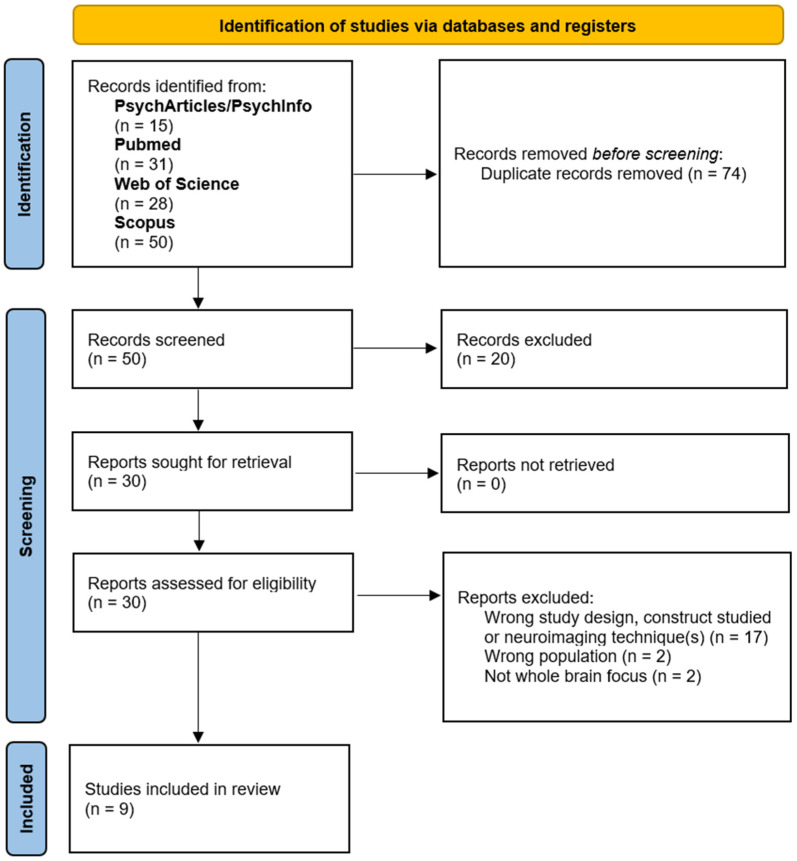
PRISMA flow diagram reporting the literature search results and study selection process.

The electronic searches identified 124 records. After removal of duplicates, 50 unique documents were screened for inclusion by the reviewers. Of these, 30 were selected for screening based on full texts. The criteria were then used to screen the full texts in a consecutive manner, such that the records not removed after evaluation based on criterion I would be evaluated using criterion II, and so forth. After full-text-based screening, twenty-one records were removed, leading to nine articles being ultimately included [4,6,15,32,33,34,35,36,37]. These articles contain a total of twelve meta-analyses on AM, EAM, and SAM. Figure 1 shows the PRISMA flow diagram detailing the literature search results and study selection process [38].

### 3.2. Charted Results of Individual Sources of Evidence

The charted data are organized in a series of tables. Table 1 contains an initial overview of the articles included in this scoping meta-review. The author(s) name(s), year, neuroimaging technique(s) used in the single studies included in the meta-analyses, memory construct under investigation, and total number of participants of the single studies included in the meta-analyses were reported. The first column links each article to an identifier (ID), also used in the subsequent tables and later in the main text to refer to each document. Each of three additional tables (Table 2, Table 3 and Table 4) gathers meta-analyses focused on one of the memory constructs under investigation (AM, EAM, and SAM). Table 2 focuses on AM, Table 3 on EAM, and Table 4 on SAM. Note that this subdivision reflects the theoretical perspective described in the Introduction and the labels found in the articles: in the next section, an argument will be presented to highlight the problems of this notation. In Table 2, Table 3 and Table 4, the experimental and control conditions of the single studies included in the meta-analyses are reported, together with the meta-analytic method adopted, the neural template used—i.e., Talairach coordinates (TAL) or Montreal Neurological Institute coordinates (MNI), and the brain activations reported in the meta-analyses.

Considering each construct separately, the neuroimaging data collected from the meta-analyses are far from uniform and show considerable differences: although some areas appear more frequently and with higher activation volume, many appear only seldom. Some of the factors that could explain this inhomogeneity are differences in the type and number of contrasts included in each meta-analysis, with differences in the experimental (target) conditions and/or control conditions. The experimental (target) conditions include cued recall (with several cueing techniques) or spontaneous recall, focusing on visual, auditory, or olfactory information. In the inherently subtractive logic of neuroimaging [21], several different control (baseline) conditions are contrasted with experimental conditions to highlight relevant areas differentially activated in the experimental condition relative to the control condition. The control conditions found in the meta-analyses include tasks that are significantly different from the experimental tasks in that they are non-memory tasks, such as rest or distracting tasks, and memory tasks, which can be relatively less similar to the experimental task such as semantic memory tasks contrasted with EAM recall, or gradually more and more similar, reaching up to contrasts between different facets of the same construct (e.g., recent vs. remote EAM). Although this leads to discontinuity in activation patterns, it is generally recognized that examining many contrasts is pivotal for exploring different facets of a construct and avoiding the loss of activations due to masking effects (e.g., [39]). Several additional sources of variability specific to each construct are explored below in Section 4 (“Discussion”). Concerning the meta-analytic methods used, the earliest paper [32] used the effect-location method [40], whereas all other meta-analyses used different versions of the Activation Likelihood Estimation (ALE) method [41,42,43]. The results derived from these variants of the ALE algorithm are considered comparable [43].

### 3.3. Analysis and Synthesis of the Results

The individual sources of evidence were examined and compared to further address the research questions. In the following subsections, the analysis process is described, together with emerging methodological, experimental, and theoretical implications. First, how the data inform the relation between memory constructs is discussed. Afterwards, separate subsections go into detail about each construct.

#### 3.3.1. The Relation Between AM, EAM, and SAM

Addis et al. [16] noted that the question of what constitutes autobiographical memory has long been debated: many researchers distinguish between episodic and semantic aspects (e.g., [3,5,13,15,17,18,19,20]), whereas many others view it as a distinctively episodic type of memory for personally experienced events that occurred at a specific time and place (e.g., [4,6,33,34,37]). The coexistence of these two definitions persists to this day and partly explains the ambiguity of AM, EAM, and SAM.

Although they appear in the literature as three memory constructs, only two broad categories of experimental (target) tasks are found in the meta-analyses: one is focused on personally experienced events, specific in time and place; and the other is focused on the retrieval of semantic personal information. When comparing AM and EAM meta-analyses, the experimental conditions are actually of the same episodic category, and many of the same studies are included in both, being labeled somewhere as ‘AM retrieval’ and elsewhere as ‘EAM retrieval’, but the target task remains the same. None of the AM meta-analyses focused on semantic personal information. The differences in labeling can then be attributed to the two different theoretical definitions of AM, since both AM meta-analyses and EAM meta-analyses we found actually studied the same episodic type of memory, leading to ambiguity and ultimately to a mismatch of constructs.

To avoid confusion and improve comparability between studies, we describe a recommendation based on experimental practices that could solve some of the problems between theories and experiments by homogenizing and systematizing the notations. We suggest considering “EAM” as the memory for autobiographical episodes. To be consistent with experimental practice, the term “AM” might appropriately encompass both EAM and SAM, but it should be noted that AM would consist of the union of EAM and SAM and would not be a separate stand-alone construct. Indeed, in this conceptualization of AM, there is no separate experimental task for AM as it is a parent construct that encompasses episodic and semantic aspects, each investigated with its own (set of) experimental task(s).

#### 3.3.2. Brain Regions Associated with EAM

In line with our proposed notation based on experimental practices, at this step of the analysis, we decided to group meta-analyses that originally reported on “AM” and “EAM” into a single group labeled “EAM”. Since all papers reported information on the lateralization (left, right, or bilateral) of activated areas, except for the earliest meta-analysis produced [32], we decided to exclude the latter from this analysis in order to distinguish the areas based also on the lateralization of activation. As discussed in Section 3 (“Results”) and shown in Table 2 and Table 3, the neurological data show a consistent degree of variability, indicating a complex and extensive network of areas. Some of these areas are frequently reported with a large number of activated voxels, but the vast majority are reported inconsistently, both in terms of the frequency of appearance and volume of activation. In the nine different meta-analyses on EAM included in this analysis, which collectively reported 50 unique activated areas, more than half (28) appeared in only one of the papers—to avoid confusion, we also consider “areas” different in terms of laterality here (e.g., activation of the right posterior cingulate cortex (PCC) is considered a different area than the left PCC or bilateral PCC). This diversity and/or inconsistency is most likely due to the wide variety and complexity of functions involved in EAM, which are studied in a variety of neuroimaging contrasts with different tasks and which may elicit very different experiences in rememberers. These and other features of the collected data (see below about reports on voxel counts) led us to choose a robust analysis method that is less sensitive to the high variability of the data. The analysis method is based on the ranking of activation areas reported by the meta-analyses.

In order to identify and classify the main areas of activation associated with EAM (differentiated by left, right, or bilateral activation), we had two types of information at our disposal:

How many activated voxels were related to a specific brain area, per meta-analysis;

How many times a brain area was involved in a specific task, across meta-analyses.

We chose not to compare voxels directly between meta-analyses because of differences in the calculation of voxel sizes and because, in some cases, exact voxel quantity related to single areas was unavailable. In those cases, the reported voxel quantities referred to clusters of different areas without reporting the contributions of individual areas in terms of voxels. For these reasons, we assessed too many risks of bias that could affect our limited analysis capabilities. Therefore, we opted for a more robust method, involving ranking areas based on each meta-analysis. Voxel counts were not used to compare areas between meta-analyses but to rank areas for each individual meta-analysis. In the case of voxel counts relating to clusters of areas, we divided the total number of voxels by the number of areas included in the cluster.

After obtaining a ranking of areas for each individual meta-analysis, we calculated the mean rank (MR) across all meta-analyses for each area of activation. Calculating the MR of each activated area across the meta-analyses allowed us to produce a ranked classification of activated areas, along with the frequency of appearance (FoA) of each area across the meta-analyses, shown in Table 5. As we aimed to identify the activated areas on which at least some of the nine meta-analyses on EAM agreed, we set a threshold requiring at least two meta-analyses reporting each area.

**Figure 2:**
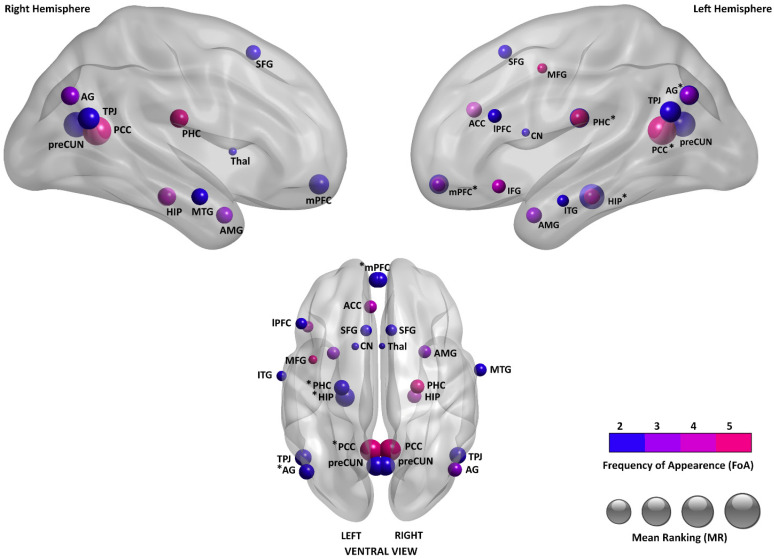
EAM-related areas of activation based on the ranked classification displayed in Table 5.

The activations shown in Table 5 were used to create Figure 2. Higher volumes correspond to higher mean ranks (MR), whereas dyes distinguish areas that were reported more or less frequently (FoA). Areas that were reported with both monolateral activation (left or right) and bilateral activation are indicated by asterisks to prevent monolateral activations from being masked by bilateral activation of the same area.

In Section 4 (“Discussion”), these data are discussed with further details and compared with the literature.

#### 3.3.3. Brain Regions Associated with SAM

Only two meta-analyses of SAM appear to be available (Document 5 [15]; Document 7 [35]) (see Table 1 and Table 4). The neural activity associated with the SAM construct presented in the two documents is not directly comparable because, as shown in Table 4, the target task conditions collected for the meta-analyses differ considerably. In Bréchet and colleagues [35], the target tasks included in the meta-analysis comprised the retrieval of general personal events, personal information, and also self-trait judgments; differently, in Martinelli and colleagues [15] the target tasks included only the retrieval of general personal events and personal information, excluding self-trait judgments. This difference is important because Martinelli et al. grouped tasks on self-trait judgments to study a completely different construct called conceptual self (CS), which concerns even more abstract information about the self, such as self-representations and knowledge of personal traits. The authors’ findings support the effectiveness of the latter subdivision, which separates less abstract semantic autobiographical knowledge such as repeated events (SAM) from more abstract personal information such as self-knowledge of personality traits (CS).

Interestingly, the list of studies included in Document 7 for SAM perfectly matches the studies collected for SAM together with the studies for CS in Document 5; moreover, the two articles used the same meta-analytic procedure, namely the ALE method. Under these conditions, a direct comparison of results shows the difference in gathering these types of tasks and contrasts under one construct (SAM) or separating them under different constructs (SAM and CS). The neural data reported in Document 7 (all studies pooled) showed scarce results for SAM, limited to anterior cingulate cortex (ACC) and PCC activations, whereas Document 5 (studies split by two constructs) showed distinct and more populated activations for SAM and CS. Namely, in Document 5, SAM was associated with PCC, ACC, middle frontal gyrus (MFG), thalamus (Thal), middle temporal gyrus (MTG), inferior frontal gyrus (IFG), superior temporal gyrus (STG), fusiform gyrus (FFG), and parahippocampal cortex (PHC) activation; whereas CS with associated with MFG, ACC and superior frontal gyrus (SFG) activation.

**Figure 3:**
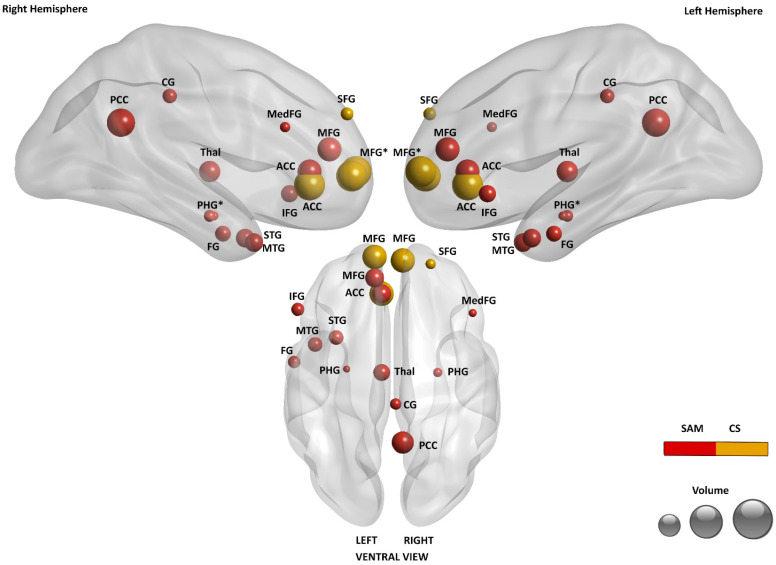
Brain areas related to SAM and CS (based on the data published by Martinelli et al. [15]).

In Figure 3, the activations reported in Document 5 for SAM and CS are depicted [15]. As noted by the authors, a significant shift from posterior to anterior structures is visible when the level of abstraction increases. This is also consistent with the results on EAM depicted in Figure 2 when considering the increase in abstraction associated with the sequence EAM–SAM–CS.

As in Figure 2, areas reported with both monolateral activation (left or right) and bilateral activation are marked with asterisks to prevent monolateral activations from being masked by bilateral activation of the same area.

## 4. Discussion

This scoping meta-review shows that multiple definitions of AM coexist in the contemporary literature. While many researchers refer to AM as encompassing episodic and semantic aspects [3,5,13,15,16,17,18,19,20], others consider it a distinctively episodic type of memory (e.g., [4,6,33,34,37]).

As mentioned above, experimental practices provide a guide to minimizing ambiguity and homogenizing and systematizing notations. Two categories of experimental retrieval tasks were used on the meta-analyses: one focuses on the retrieval of events, the other on the retrieval of semantic autobiographical information. “AM retrieval” tasks and “EAM retrieval” tasks are indistinguishable throughout the meta-analyses and focus on events; therefore, autobiographical memory of episodes (called “AM” in some papers and “EAM” in others) can be uniformly considered EAM. To be consistent with the experimental practice, the term “AM” may denote a construct that encompasses both EAM and SAM, not a distinct construct identified by a separate experimental task (which does not exist).

Moreover, episodic and semantic components do not function in isolation from each other, e.g., participants often search general autobiographical knowledge to retrieve an episode, or episodes may be complemented by general representations that may relate to the environment or some recurring features [5,32]. Such coordination may suggest that EAM and SAM identify a continuum on the episodic–semantic axis [3,44,45]. Recently, Addis and Szpunar [46] proposed surpassing the idea of a single-dimension axis in favor of a three-dimensional model based on memory content (conceptual vs. perceptual), temporal specificity (general vs. specific), and self (shared vs. idiosyncratic).

The semantic aspects of autobiographical memory seem to be far less studied than the episodic aspects. We compared the only two meta-analyses on SAM available to date, which use different definitions of SAM: one refers to the entire semantic continuum [35], while the other divides this continuum with two constructs (i.e., SAM and CS; [15]). As shown in Section 3 (“Results”), the findings of Martinelli and colleagues seem to be more convincing, for two main reasons. The first is that the differentiation between SAM and CS is more consistent with the scientific literature, as these constructs are considered different and partially independent in that the latter is devoid of the spatiotemporal context of acquisition [2,45,47]. The second refers to neuroimaging results: Martinelli et al. [15] included 13 studies on SAM and 12 studies on CS, whereas Bréchet and colleagues [35] used the same 13 and 12 studies together under one same construct (SAM). As a result, Martinelli et al. identified two distinct and populated activation patterns for SAM and CS, whereas Bréchet and colleagues found scarce results for their definition of SAM, in contrast with the literature (e.g., [48,49]). ALE meta-analyses aim to identify above-chance convergence in studies investigating the same construct by pooling the 3D activation coordinates reported in the studies [21,45]. It appears that dividing the semantic gradient into less abstract information (SAM) and more abstract information (CS) reveals the convergence of many more activations rather than pooling all the studies together irrespective of the abstraction level. Moreover, CS is studied with a distinct neuroimaging contrast (knowledge of self vs. other people traits), and it is different from SAM as it sits at the interface between the concepts of memory and self, in that it covers abstract information about the self, such as self-representations and knowledge of personal traits [2,47,50] and has neural mechanisms distinct from autobiographical facts [45,51].

On the other side of the abstraction axis, on the relationship between EAM and SAM, additional distinctions are suggested by some authors in the semantic gradient, such as dividing autobiographical facts from repeated events, as the latter ones show activations much more similar to EAM than autobiographical facts (e.g., [45,52]). These possible further distinctions in SAM may help better describe the substantial overlap between EAM and SAM (see Figure 2 and Figure 3).

The literature on EAM is far more extensive than on SAM. EAM appears to be a complex and multifaceted construct, such that striking abundance and variability can be found in the meta-analyses with regard to the areas of activation. It is interesting to note that the agreement between the meta-analyses is quite limited. Possible factors for this variability can be attributed to the multifaceted nature of EAM and the many different methodological strategies used to highlight activations. Different activations may emerge depending on the types and proportions of contrasts that are pooled from single studies (e.g., remote vs. recent memories [36]; younger vs. older rememberers [37]; and EAM retrieval vs. non-memory, semantic memory, laboratory memory, or rest [6]). However, our analysis shows that some brain areas emerge as more activated in terms of volume and are more frequently reported in meta-analyses. The MR is an index of the magnitude of the average reported activation volume in relation to other areas, whereas the FoA is an index of how frequently an area is reported in meta-analyses.

As shown in Table 5, the brain area presenting the highest mean rank among the meta-analyses on EAM is the PCC, displaying both bilateral activation (MR = 1.2; FoA = 5) and monolateral left activation (MR = 1.33; FoA = 3). This area remains, to date, one of the least understood among cerebral cortices [4,53,54], although its high connectivity and metabolism suggest an important cognitive role [32,55]. This role is difficult to disentangle because PCC activation is present in a large number of cognitive processes, mainly with synchronous activations of many different brain areas, since the PCC is part of several different brain networks (e.g., [54]). PCC activation is related to “being caught up in” mental content, particularly referring to self-referential processing but also social cognitive processing, the disruption of attention on tasks, and craving. On the other hand, PCC deactivation is associated with present-centered attention or awareness [6,54,56]. Leech and Sharp [55] focused on the role of the PCC in attention, dividing it into functionally separate ventral and dorsal regions: while activity in both regions was dependent on arousal, activation patterns changed with different combinations of internal vs. external and narrow vs. broad attentional focus. In a recent paper, Foster et al. [53] proposed a tripartite model of the PCC, where the dorsal portion is linked with executive and decision-related processes across both mnemonic and non-mnemonic tasks, the ventral portion is associated with predominantly mnemonic processes and information related to the context/environment, and the retrosplenial portion is tied to spatial memory and navigation. As these three sub-regions cooperate, the collective PCC is reported to process and integrate internal information, including but not limited to autobiographical material, to supply ongoing processes such as learning and decision-making [53].

The left hippocampus (HIP) and bilateral precuneus (PreCUN) show the second highest MR (1.5). The HIP is largely connected with the PCC and their patterns of activation are different for recent or remote memories [36] as well as for younger or older rememberers [37]. HIP activation is largely more prominent in EAM rather than in SAM or in retrieval of general semantic knowledge [15]; this is consistent with its proposed roles encompassing the collection of sensory and perceptual details and the following construction of mental images and representations that are coherent in their spatiotemporal context [57,58,59,60]. As the HIP binds together details to build representations, the PHC (MR = 3) provides it with pieces of contextual associations necessary for their construction [60,61]. The PreCUN is associated with episodic memory, visuospatial imagery, and self-referential processing; the latter mainly encompasses the experiencing of first-person perspective and agency [62,63].

The temporo-parietal junction (TPJ) (MR = 2.5) contributes to a series of functions such as memory, attention, and language, particularly in the context of social cognition [64]. With the distinction and integration of self-related and other-related representations, it allows us to build the social context of mental representations [65]. The angular gyrus (AG, MR = 3), an anatomical neighbor to the TPJ, shares participation with it on several functions (e.g., [64]). Integrating attention and memory functions, with its strong connectivity with the HIP, the AG monitors retrieval operations and integrates perceptual details to convey vivid memories [66,67].

The medial prefrontal cortex (mPFC) (MR = 4.67), most notably its anterior part (MR = 3), appears as an important area for EAM. Communicating especially with the HIP, it has been proposed to register associations linking spatial, temporal, and contextual information with responses shown to be adaptive, while also mediating the corresponding emotional, social, and self-related information [68,69,70]. As one of three subdivisions of the mPFC, the anteromedial prefrontal cortex (amPFC) is reported to be specialized in managing self-related and affective processing [6,71]. The neighboring ACC (MR = 4.75) participates in emotional processing, including emotion regulation and valence [68,72].

In Table 5, additional secondary areas that present a MR ≥ 5 are listed. These include the amygdala (AMG); several activations pertaining to the temporal (middle temporal gyrus, MTG; inferior temporal gyrus, ITG), frontal (superior frontal gyrus, SFG; inferior frontal gyrus, IFG; middle frontal gyrus, MFG), and prefrontal cortexes (lateral prefrontal cortex, lPFC); and, finally, small activations related to the CN and thalamus.

### Limitations and Future Directions

The methodology used in this research contribution is a flexible and powerful instrument that has proved to appropriately address the research questions. However, different methods would be required to further explore some specific aspects. For example, only qualitative evaluations could be made about the reasons underlying the high degree of variability present in the neuroimaging data reported by the meta-analyses. The quantitative impact of each source of variability (e.g., different meta-analyses including different neuroimaging contrasts) would be an interesting and useful theme to investigate but could not be assessed in this study.

A limitation of this scoping meta-review is the potential redundancy arising from overlap of primary studies included in the various meta-analyses analyzed. This overlap may have inflated the frequency counts of certain brain regions, potentially overemphasizing findings derived from original studies included in multiple reviews. Future research could benefit from implementing advanced meta-analytic techniques capable of modeling dependence structures among overlapping datasets to provide more precise estimates of neural correlates.

Another limitation concerns the scarcity of meta-analytic studies on SAM. The only two available meta-analyses were also based on different definitions of SAM. So, we found interesting insights comparing the two and showed that one definition appears more appropriate than the other, but we could not compare multiple meta-analytic results based on one same SAM definition.

We focused on how the activations related to the memory constructs appear in the normative, healthy population. Of course, a whole other dimension opens up when one considers the many different existing clinical populations and lesion studies. Many incidents and disorders have an impact on the memory constructs that we studied here, but not many have received meta-analyses yet—e.g., Thome et al. [73]’s study about posttraumatic stress disorder.

Our scoping meta-review focused on activation patterns derived from PET and fMRI studies, and this decision was based on the prevalence and established use of these methodologies. Other neuroimaging techniques (e.g., functional Near-Infrared Spectroscopy, fNIRS; electroencephalogram, EEG) were out of the scope of the present work mainly for reasons of comparability across results. Also, analyses of brain connectivity and its association with behavioral data represent important complementary approaches. Some of the included meta-analyses and the related literature suggest that networks such as the default mode network and hippocampal–angular gyrus connectivity play key roles in autobiographical memory retrieval and are linked to behavioral measures like memory specificity and recollection accuracy. However, systematic examination of connectivity and direct brain–behavior correlations was beyond the scope of the present review and represents a promising avenue for future research.

## 5. Conclusions

This scoping meta-review showed that the literature on autobiographical memory and related constructs is currently fragmented around different construct definitions. Concerning AM and EAM, we proposed the homogenization of notations based on experimental practices, considering all contrasts that focus on autobiographical episodes as experimental (target) task to be studying EAM, regardless of it being called “AM” or “EAM” in the original papers. In this perspective, EAM and SAM taken together may constitute AM, which does not have a separate experimental task and brain activation pattern, and would therefore indicate nothing but an array of its sub-constructs. Concerning SAM, we found only two meta-analyses that also use different definitions of the construct. The operationalization of SAM used by Martinelli and colleagues [15] is the most promising of the two, distinguishing between SAM and CS. Unfortunately, however, these constructs are currently far less studied compared to EAM.

Applying the notations just discussed, we found an abundance of neuroimaging data on EAM. Our data analysis showed that a large number of areas of activation in the brain has been reported, and the ten areas of activation that are currently reported with the highest volume across meta-analyses are the bilateral PCC, left PCC, left HIP, bilateral PreCUN, bilateral TPJ, left AG, bilateral amPFC, left PHC, bilateral AG, and bilateral HIP. However, the meta-analyses show high variability and limited agreement between sources. The multitude of different activation patterns that can be associated with EAM certify that the nature of this construct is complex. Future models of EAM aiming for comprehensiveness will need to manage the sources of variability in the literature: while some of these sources could possibly be controlled using caution and increasingly refined analysis methods, some others characterize the very nature of this inherently multifaceted construct and are reflected in all of the different experiences that characterize EAM in our daily lives.

## Figures and Tables

**Table 1:** Overview of the articles included in the present study.

ID	Author(s)	Year	Neuroimaging Technique(s)	Memory Construct(s)	Total Number of Participants
1	Svoboda et al. [32]	2006	PET, fMRI	AM	243
2	McDermott et al. [4]	2009	PET, fMRI	AM	n/a
3	Spreng et al. [33]	2009	PET, fMRI	AM	228
4	Kim [6]	2012	PET, fMRI	AM	494
5	Martinelli et al. [15]	2013	PET, fMRI	EAM, SAM	153, 186
6	Kim [34]	2015	PET, fMRI	AM	656
7	Bréchet et al. [35]	2018	PET, fMRI	EAM, SAM	813, 396
8	Boccia et al. [36]	2019	fMRI	EAM	1409
9	Fenerci et al. [37]	2022	PET, fMRI	AM	656

n/a: not reported.

**Table 2:** Brain area activations related to AM.

ID	Experimental Conditions (AM Retrieval)	Control Conditions (Baseline)	Procedure	Template	Brain Areas
1	AM recall (not specified if cued or free)	AM retrieval Distracting non-memory tasks Resting state Semantic memory retrieval	“effect-location” method	TAL	mPFC; vlPFC; OFC; FEF; M1; ACC; RSC; PCC; PreCUN; TPJ; MTL; FFG; TL; INS; OL; AMG; BG; Thal; Brainstem; CBM.
2	Cued recall (visual; auditory; true/false judgments)	Distracting nonmemory tasks Resting state Semantic memory retrieval	ALE method	MNI	Left mPFC; Left ACC; Left PMC; Left IFG; Left MFG; Bil AG; Bil PHC; Right aHIP; Left PCC; Thal; CN.
3	Cued recall (visual; auditory; true/false judgments)	Distracting nonmemory tasks Semantic memory retrieval	ALE method	TAL	Bil preCUN; Bil PCC; Bil HIP; Bil PHC; Bil AMG; Bil TPJ; Left mPFC; Left ACC; Left STS; Left MTG; Left ITG; Bil vlPFC; Bil TL; Bil MFG; Left lPFC; Left FP; Right TP; Right STS; Right MTG; Left TP; Left OL; Left dlPFC; Right Thal; Left SFG.
4	Cued recall (visual; auditory; true/false judgments) AM visualization Free AM retrieval	Distracting nonmemory task Laboratory-based episodic memory retrieval Resting state Semantic memory retrieval	ALE method	TAL	Bil amPFC; Left SMA; Left MFG; Left IFG; Bil INS; Bil HIP; Bil PHC; Bil AMG; Bil LTC; Bil PCC; Left IPL; Right SOG; Left Thal; Left CN.
6	Cued recall (visual; auditory) Free AM retrieval	Distracting nonmemory task Laboratory-based episodic memory retrieval Resting state Semantic memory retrieval	ALE method	TAL	Bil PCC; Bil RSC; Bil AMG; Bil HIP; Bil PHC; Bil AG; Bil amPFC; Left aLTC; Bil SFG; Left IFG; Left MFG; Right Thal; Left CN.
9	AM recall (not specified if cued or free)	AM retrieval Distracting nonmemory tasks Resting state Semantic memory retrieval	ALE method	MNI	----younger---- Left PCC; Left HIP; Left AG; Left mPFC; Left lPFC ----older---- Bil HIP; Bil AG; Right MTG; Left PCC; Bil mPFC.

Abbreviations: ACC = anterior cingulate cortex; AG = angular gyrus; aHIP = anterior hippocampus; aLTC = anterior lateral temporal cortex; AMG = amygdala; amPFC = anteromedial prefrontal cortex; BG = basal ganglia; Bil = bilateral; CBM = cerebellum; CN = caudate nucleus; dlPFC = dorsolateral prefrontal cortex; FEF = frontal eye fields; FFG = fusiform gyrus; FP = frontal pole; HIP = hippocampus; IFG = inferior frontal gyrus; INS = insula; IPL = inferior parietal lobule; ITG = inferior temporal gyrus; lPFC = lateral prefrontal cortex; LTC = lateral temporal cortex; MFG = middle frontal gyrus; MNI = Montreal Neurological Institute coordinates; mPFC = medial prefrontal cortex; MTG = middle temporal gyrus; MTL = medial temporal lobe; M1 = primary motor cortex; OFC = orbitofrontal cortex; OL = occipital lobe; PCC = posterior cingulate cortex; PHC = parahippocampal cortex; PMC = premotor cortex; PreCUN = precuneus; RSC = retrosplenial cingulate cortex; SFG = superior frontal gyrus; SMA = supplementary motor area; SOG = superior occipital gyrus; STS = superior temporal sulcus; TAL = Talairach coordinates; Thal = thalamus; TL = temporal lobe; TP = temporal pole; TPJ = temporoparietal junction; vlPFC = ventrolateral prefrontal cortex.

**Table 3:** Brain area activations related to EAM.

ID	Experimental Conditions (EAM Retrieval)	Control Conditions (Baseline)	Procedure	Template	Brain Area
5	EAM recall (not specified if cued or free)	Laboratory-based episodic memory retrieval Memory tasks Semantic memory retrieval	ALE	TAL	Bil PHC; Bil CBM; Left HIP; Bil preCUN; Left MTG; Right PCC; Left MFG.
7	EAM recall (not specified if cued or free)	Distracting nonmemory tasks Memory tasks Resting state Semantic memory retrieval	ALE	MNI	Bil TPJ; Bil PCC; Left ACC; Left PHC; Left ITG; Left IFG; Bil SFG; Left MFG.
8	Cued recall (auditory; visual; olfactory) Generation of mental images True/false judgements	Distracting nonmemory tasks Laboratory-based episodic memory retrieval Other EAM conditions (specific vs. general; recent vs. remote; and vivid vs. nonvivid) Semantic memory retrieval	ALE	MNI	Bil PCC; Left PHC; Left AG; Right PHC; Left ACC; Left vmPFC; Right AG; Right CBM; Right MTG.

Abbreviations: vmPFC = ventromedial prefrontal cortex. For other abbreviations see Table 2.

**Table 4:** Brain area activations related to SAM.

ID	Experimental Conditions (EAM Retrieval)	Control Conditions (Baseline)	Procedure	Template	Brain Area
5	SAM recall (not specified if cued or free): retrieval of general personal events or personal information, no self-trait judgments	Laboratory-based episodic memory	ALE	TAL	Right PCC; Left ACC; Bil MFG; Thal; Left STG; Left MTG; Left IFG; Left FFG; Bil PHC.
7	SAM recall (not specified if cued or free): retrieval of general personal events; personal information or also self-trait judgments	Nonpersonal semantic memory	ALE	MNI	Bil ACC; Bil PCC.

Abbreviations: STG = superior temporal gyrus. For other abbreviations see Table 2.

**Table 5:** Ranked classification of activated areas for EAM. The mean rank and frequency of appearance are calculated across meta-analyses.

Mean Rank (MR)	Frequency of Appearance (FoA)	Area Name and Laterality
1.2	5	Bil PCC
1.33	3	Left PCC
1.5	2	Left HIP
1.5	2	Bil PreCUN
2.5	2	Bil TPJ
3	2	Left AG
3	2	Bil amPFC
3	2	Left PHC
4	3	Bil AG
4	4	Bil HIP
4	5	Bil PHC
4.67	3	Left mPFC
4.75	4	Left ACC
5	3	Bil AMG
5	2	Right MTG
5.5	2	Bil SFG
6.25	4	Left IFG
6.5	2	Left lPFC
7	2	Left ITG
8	5	Left MFG
10	2	Left CN
11	2	Right Thal

For abbreviations, see main text and Table 2.

## Data Availability

No new data were created or analyzed in this study. Data sharing is not applicable to this article.

## Supplementary Materials

The following supporting information can be downloaded at: https://www.mdpi.com/article/10.3390/brainsci15050515/s1, Table S1: Preferred Reporting Items for Systematic reviews and Meta-Analyses extension for Scoping Reviews (PRISMA-ScR) Checklist.

## Appendix A

**Table A1:** Individual Database Searches.

Date	Database	Query	Results
3 May 2023	PsycInfo/PsycArticles	AB autobiogr* AND AB meta-analys* AND AB (imaging or fmri)	13
3 May 2023	PsycInfo/PsycArticles	AB autobiogr* AND AB meta-analys* AND AB (positron or pet)	2
3 May 2023	PubMed	((“autobiogr*”[Title/Abstract]) AND (meta-analys*[Title/Abstract])) AND (imaging or fmri[Title/Abstract])	29
3 May 2023	PubMed	((“autobiogr*”[Title/Abstract]) AND (meta-analys*[Title/Abstract])) AND (positron or pet[Title/Abstract])	2
3 May 2023	Web of Science (all databases)	AB = (autobiogr* AND meta-analys* AND (imaging or fmri))	28
3 May 2023	Web of Science (all databases)	AB = (autobiogr* AND meta-analys* AND (positron or pet))	0
3 May 2023	Scopus	(TITLE-ABS-KEY (autobiogr*) AND TITLE-ABS-KEY (meta-analys*) AND TITLE-ABS-KEY (imaging OR fmri))	42
3 May 2023	Scopus	(TITLE-ABS-KEY (autobiogr*) AND TITLE-ABS-KEY (meta-analys*) AND TITLE-ABS-KEY (positron OR pet))	8
